# Increased Rates of Infectious Diseases in Fibromyalgia Patients: A Population-Based Case-Control Study

**DOI:** 10.3390/biomedicines12122821

**Published:** 2024-12-12

**Authors:** Michal Vinker-Shuster, Eli Magen, Ilan Green, Eugene Merzon, Avivit Golan-Cohen, Ariel Israel

**Affiliations:** 1 Department of Pediatrics, Assuta Ashdod University Medical Center, Ashdod 7747629, Israel; 2 Faculty of Health Sciences, Ben-Gurion University of the Negev, Beer Sheva 8410501, Israel; allergologycom@gmail.com; 3 Leumit Health Services, Tel Aviv-Yafo 6473817, Israel; igreen@leumit.co.il (I.G.); emerzon@leumit.co.il (E.M.); agolanchoen@leumit.co.il (A.G.-C.); aisrael@leumit.co.il (A.I.); 4 Medicine A Department, Assuta Ashdod University Medical Center, Ashdod 7747629, Israel; 5 Department of Family Medicine, Sackler Faculty of Medicine, Tel-Aviv University, Tel Aviv-Yafo 6997801, Israel; 6 Adelson School of Medicine, Ariel University, Ariel 4070000, Israel

**Keywords:** fibromyalgia, immune system disease, infections, epidemiology

## Abstract

**Introduction**: Fibromyalgia (FM) patients are known to have medical comorbidities. This study characterized the rates of infectious diseases in FM patients compared to the general population. **Methods**: A nationwide population-based case-control study was conducted, including all patients diagnosed with FM by a rheumatologist compared to a matched 5:1 control group within a large health maintenance organization in Israel (January 2002 to December 2023). Demographic, anthropometric, and health habit data were extracted from medical records as well as the ICD-9 codes of diagnoses related to infectious diseases in 9232 FM patients and 46,160 controls. Infection rates in the FM patients were compared to the controls over a mean follow-up of 6.7 years. **Results**: The FM patients had a significantly higher incidence of viral, bacterial, fungal, and parasitic diseases compared to the controls. The FM patients had significantly higher odds ratios (ORs) for respiratory/sinopulmonary infections, including upper respiratory tract infections (OR = 1.49), *influenza* (OR = 1.80), tonsillitis (OR = 1.40), sinusitis (OR = 1.98), otitis media (OR = 1.84), otitis externa (OR = 1.48), and pneumonia (OR = 1.60), all *p* < 0.01. They also experienced more gastrointestinal infections, including gastroenteritis (OR = 1.40), *Helicobacter pylori* (OR = 2.05), *candidal* esophagitis (OR = 7.88), and *giardiasis* (OR = 3.41), all *p* < 0.01. They had a higher prevalence of genitourinary infections, including urinary tract infections (OR = 1.79) and pelvic inflammatory disease (OR = 3.17), *p* < 0.01 as well as skin infections such as abscess (OR = 1.74) and cellulitis (OR = 1.64) and systemic infections such as symptomatic COVID-19 (OR = 1.76) and *Cytomegalovirus* (*CMV*) (OR = 1.85), all *p* < 0.01. **Conclusions**: The FM patients had a significantly higher incidence of infectious diseases than the general population. Further research is needed to better understand the underlying mechanisms and develop targeted interventions to address infection risks in FM patients.

## 1. Introduction

Fibromyalgia (FM) is a chronic condition characterized by widespread musculoskeletal pain. The prevalence of FM is estimated to be 2.7% in the general population, with a female predominance [1]. While pain is the primary symptom, patients with FM may also experience cognitive impairment, fatigue, and sleep and mood disorders.

The etiology of FM remains unknown [2] but is considered multifactorial, involving a genetic predisposition and emotional, cognitive, and inflammatory factors [3]. Although these patients’ complete blood count (CBC), erythrocyte sedimentation rate (ESR), and C-reactive protein (CRP) are normal, and tissue examinations do not indicate inflammation, the recent literature suggests the presence of inflammatory or autoimmune mechanisms [1,4,5]. Wallace et al. [6] identified a unique profile of pro-inflammatory cytokines and chemokines in FM patients, including interleukin (IL)-5, IL-6, IL-8, and interferon-gamma. In animal models, transferring immunoglobulin G (IgG) from FM patients to mice induced sensory hypersensitivity [7]. Neuroinflammation has been identified in the dorsal root ganglia, mediated by polymorphonuclear neutrophils [2]. Caro et al. found that FM patients had significantly lower mean levels of immunoglobulins than healthy individuals [8].

Although there is scant literature on the prevalence of infectious diseases in FM patients, a recent meta-analysis reported a higher standard mortality ratio (SMR) of 1.66 for deaths due to infections, including pneumonia, septicemia, and others in FM patients [9]. Other studies have found higher risks for sinopulmonary infections [8] and periodontal abscess in FM patients [10].

To better understand these issues, this study characterized the rates of infectious diseases in patients with fibromyalgia (FM) to determine to what extent they are at greater risk of infections than the general population.

## 2. Methods

A population-based observational case-control study of patients with FM was conducted in Leumit Health Services (LHS), a large nationwide health maintenance organization in Israel, which provides services to approximately 750,000 enrollees. Patients aged 18 to 90 years diagnosed with FM according to the International Classification of Diseases, 9 (ICD-9) codes who had at least three rheumatologist visits between January 2002 and December 2023 were included in the study. Data were extracted up to March 2024.

The FM group was defined as patients with FM, and the baseline index date was defined as the FM diagnosis. A 5:1 ratio matched for age, gender, and year of the first LHS membership control group was randomly selected from the study population. Five control individuals with birth dates closest to each case were selected. To synchronize the timelines for cases and controls, the control subjects were assigned the same index date as their matched cases. Each control patient was selected only once.

The electronic medical records for both groups included demographic, anthropometric, and health habit (smoking and physical activity) information. Socio-economic status (SES) was based on the Israel Central Bureau of Statistics classification by home address. The ICD-9 codes were analyzed for infectious respiratory, gastrointestinal, genitourinary, dermal, and systemic diseases reported after the FM diagnosis.

### Statistical Analyses

A *t*-test was used to compare continuous variables, and Fisher’s test was used to compare categorical variables. The infectious disease rates were calculated in patients with and without FM at baseline, as determined by the index date, and up to 10 years later (the mean follow-up time was 6.7 years in both groups; *p* = 0.11). A false discovery rate (FDR) analysis was employed to control for multiple comparisons in the analysis of all concomitant comorbidities, including other chronic diagnoses and chronic medical treatment. The statistical analyses were performed in R statistical software version 4.3. A *p*-value of < 0.05 was considered significant.

This study was approved by the LHS ethics committee (0011-23-LEU).

## 3. Results

Table 1 presents the demographic and clinical data for the 9232 patients with FM and the 46,160 controls. There was equal female predominance in both groups (86.8%) with no significant difference in average age or SES. The FM group had a significantly higher average BMI and smoking rate, and reported lower physical activity *(p* < 0.001). Other chronic diagnoses including headache, temporomandibular joint disorder, irritable bowel disease, chronic fatigue syndrome, depression, and anxiety were more frequent in the FM group *p* (< 0.001).

Table 2 presents the prevalence and odds ratios (ORs) for the infectious diseases identified during the 10-year period in the FM group compared to the control group. The FM patients showed a higher prevalence of respiratory tract infections, including upper respiratory tract infections (URTI) with an odds ratio (OR) of 1.49, acute bronchitis (OR 1.52), acute nasopharyngitis (OR 1.19), acute laryngopharyngitis (OR 1.25), acute tracheobronchitis (OR 1.77), acute bronchiolitis (OR 2.08), *influenza* (OR 1.8), acute tonsilitis (OR 1.4), sinusitis (OR 1.98), otitis media (OR 1.84), otitis externa (OR 1.48), and pneumonia (OR 1.60), all *p* < 0.01.

The FM patients also had a higher prevalence of gastrointestinal system infections, including gastroenteritis (OR 1.4), *Helicobacter pylori* gastritis (OR 2.05), *candidal* esophagitis (OR 7.88) and *giardiasis* (OR 3.41), all *p* < 0.01, as well as genitourinary infections, including urinary tract infections (OR 1.79) and pelvic inflammatory disease (OR 3.17), *p* < 0.01.

The FM patients also presented with a higher prevalence of skin and soft tissue bacterial infections, including abscess (OR 1.74), cellulitis (OR 1.64), and impetigo (OR 1.54) as well as fungal infections such as *Tinea pedis* (OR 1.64), *Tinea cruris* (OR 1.71), *candidiasis* (OR 1.97), *Tinea versicolor* (OR 1.87), and viral warts (OR 1.39), all *p* < 0.01.

The FM patients also exhibited higher rates of systemic viral infections such as *cytomegaloviral* disease (*CMV*) and COVID-19 and a range of systemic bacterial infections such as lymphadenitis and staphylococcus infections, *p* < 0.01.

## 4. Discussion

This population-based observational case-control study was designed to analyze the associations between FM and infectious diseases. The findings suggest that FM was associated with a higher OR for infectious bacterial, viral, fungal, and parasitic diseases. These results are consistent with a recent meta-analysis indicating a higher mortality rate (1.66-fold) from infections such as pneumonia, septicemia, and others in FM patients compared to the general population [9]. Other studies have also reported a higher prevalence of sinopulmonary infections [8] and periodontal abscesses [10] in comorbid FM.

The higher rate of infectious disease diagnoses in fibromyalgia patients may have been influenced by an ascertainment bias, since these patients tend to have more frequent physician visits, undergo more testing, and report symptoms more often. In this cohort, in accordance with the literature, patients with FM had higher rates of headaches and chronic fatigue syndrome and were more likely to have psychiatric conditions such as depression and anxiety. These factors may contribute to detection bias and an increased likelihood of infectious disease diagnoses. Another possible explanation is their relatively unhealthy lifestyle, that includes higher rates of smoking and obesity.

However, this increased susceptibility to infections in FM patients may suggest an underlying form of immune dysfunction, such as a higher tendency toward neoplastic, autoimmune, and allergic diseases, as previously described [11,12]. Caro et al. found lower levels of certain immunoreactant combinations (including total IgA and its subclasses IgA1 and IgA2, IgG subclasses—IgG1, IgG2, IgG3 and IgG4, IgM, and mannose-binding lectin [MBL]). The IgA1 subclass and augmented IgG, IgM, and IgD levels protect against bacteria in the gastrointestinal tract and the naso-oro-pharyngeal area [8]. Thus, lower mean levels of immunoglobulins could potentially account for the increased vulnerability of the FM patients in this study to sinopulmonary infections (URTI, sinusitis, otitis media, tonsilitis, laryngopharyngitis, pneumonia, etc.). Another possible explanation may be related to respiratory disturbances in FM patients, as found in a recent meta-analysis [13] which reported reductions in chest expansion, maximal ventilatory volume, and maximal inspiratory and expiratory pressures in FM patients compared to healthy controls.

The higher risk for UTI found in this study could be related to the higher prevalence of overactive bladder symptoms in FM patients [14], resulting in more frequent urine cultures ordered for symptoms of urinary urgency and frequency [15]. The increased rates of skin infections, including cellulitis, abscess, and impetigo found in the FM patients could be associated with hyperhidrosis and dermatitis, which are common dermatological manifestations of FM [16].

Decreased mobility due to pain [9] and a greater tendency to seek medical care [17] for simple viral illnesses or self-limiting fever episodes could also contribute to the higher incidence of self-limited infection diagnoses in FM patients. The decreased physical activity and mobility of FM patients could also increase the risk of infections such as cellulitis and impetigo [18].

Recent studies have also pointed to the role of neuroinflammation in FM, mediated by polymorphonuclear neutrophils in the dorsal root ganglia [19]. FM patients may have abnormal immune responses in the brain, making them more susceptible to abnormal central responses to low-level immune challenges [20].

This study has several limitations. First, its population-based observational nature may have introduced biases related to data collection and the accuracy of the medical records. We do not have data regarding FM patients’ lifestyle, including eating habits, drug abuse, or alcoholism. Further prospective studies are needed to confirm the findings and explore the causal relationships between FM and increased infection rates. Further, the study population records were extracted from a single health maintenance organization in Israel, which may limit the generalizability of the findings to other populations. More research is needed to elucidate the underlying mechanisms driving the increased susceptibility to infections in FM patients. This could involve exploring the role of specific immune pathways, genetic factors, and environmental influences.

Overall, however, the findings indicate higher rates of infections in FM patients, which underscores the need for further research to better understand the underlying mechanisms governing infectious comorbidity in FM patients. Healthcare providers should be aware of the increased rates of infections in FM patients and consider implementing preventive measures. These could include regular monitoring for signs of infections and timely vaccinations.

## Figures and Tables

**Table 1:** Demographics of patients with fibromyalgia (FM) and controls.

		FM n = 9232 (%)	Control n = 46,160 (%)	*p*-Value	Odds Ratio [CI]
Gender	Female	8017 (86.8%)	40,085 (86.8%)	1.000	1.00 [0.94 to 1.07]
	Male	1215 (13.2%)	6075 (13.2%)	1.000	1.00 [0.94 to 1.07]
Age (years) mean, SD		47.6 ± 12.4	47.6 ± 12.4	0.966	
Age category	19–29	812 (8.80%)	4043 (8.76%)	0.904	1.00 [0.93 to 1.09]
	30–39	1542 (16.70%)	7740 (16.77%)	0.891	1.00 [0.94 to 1.06]
	40–49	2703 (29.28%)	13,491 (29.23%)	0.920	1.00 [0.95 to 1.05]
	50–59	2679 (29.02%)	13,447 (29.13%)	0.831	0.99 [0.95 to 1.04]
	60–69	1120 (12.13%)	5556 (12.04%)	0.793	1.01 [0.94 to 1.08]
	70–79	332 (3.60%)	1662 (3.60%)	1.000	1.00 [0.88 to 1.13]
	80–89	44 (0.48%)	221 (0.48%)	1.000	1.00 [0.70 to 1.38]
Socio-economic status (1–20)		8.53 ± 3.50	8.58 ± 3.52	0.216	
Weight (kg) *		75.4 ± 17.1	73.3 ± 16.5	0.000	
BMI (kg/m^2^) **		28.5 ± 6.1	27.6 ± 5.8	0.000	
BMI category	<18.5 Underweight	201 (2.20%)	1001 (2.22%)	0.938	0.99 [0.85 to 1.16]
	18.5–24.9 Normal	2607 (28.53%)	15,222 (33.75%)	0.000	0.78 [0.75 to 0.82]
	25–29.9 Overweight	2983 (32.65%)	15,282 (33.88%)	0.023	0.95 [0.90 to 0.99]
	≥30 Obese	3346 (36.62%)	13,602 (30.15%)	0.000	1.34 [1.28 to 1.40]
Smoking status ***	Non-smoker	6845 (74.14%)	35,773 (77.50%)	0.000	0.83 [0.79 to 0.88]
	Past smoker	171 (1.85%)	631 (1.37%)	0.001	1.36 [1.14 to 1.62]
	Smoker	1979 (21.44%)	7841 (16.99%)	0.000	1.33 [1.26 to 1.41]
Physical activity ****	1–3 h weekly	1341 (14.53%)	9244 (20.03%)	0.000	0.68 [0.64 to 0.72]
	>3 h weekly	415 (4.50%)	3144 (6.81%)	0.000	0.64 [0.58 to 0.72]
	None	4075 (44.14%)	16,551 (35.86%)	0.000	1.41 [1.35 to 1.48]
	Occasionally	3016 (32.67%)	14,681 (31.80%)	0.104	1.04 [0.99 to 1.09]
784.0 Headache		4798 (51.9%)	12,469 (27%)	0.000	2.92 [2.79 to 3.06]
524.61 Temporomandibular joint (TMJ) disorder		153 (1.6%)	257 (0.6%)	0.000	3.01 [2.44 to 3.70]
564.1 Irritable bowel syndrome		696 (7.5%)	820 (1.7%)	0.000	4.51 [4.06 to 5.01]
780.71 Chronic fatigue syndrome		121 (1.3%)	58 (0.1%)	0.000	10.55 [7.65 to 14.70]
F41.9 Anxiety disorder, unspecified		2664 (28.8%)	6331 (13.7%)	0.000	2.55 [2.42 to 2.69]
F32 Depressive episode		2178 (23.6%)	3457 (7.5%)	0.000	3.81 [3.59 to 4.05]

Fibromyalgia, FM; Body mass index. BMI; * Weight data are missing for 35 FM patients (0.38%) and 719 (1.56%) control patients. ** BMI data are missing for 95 FM patients (1.03%) and 1053 (2.28%) control patients. *** Smoking status data are missing for 237 FM patients (2.57%) and 1915 (4.15%) control patients. **** Physical activity status data are missing for 385 FM patients (4.17%) and 2540 (5.5%) control patients.

**Table 2:** Prevalence of infectious diseases in fibromyalgia (FM) patients vs. controls. False discovery rate (FDR) analysis was employed to control for multiple comparisons in the analysis of all concomitant comorbidities, including other chronic diagnoses and chronic medical treatment.

ICD-9 Codes	Diagnoses	Pathogen Type	FM n (%)	Control n (%)	*p*-Value	BH FDR	OR [CI 95%]
Respiratory System Infections							
465.9	Acute upper respiratory infections [URTI] of unspecified site	Viral	5053 (54.73)	20,668 (44.78)	<0.001	<0.001	1.49 [1.43 to 1.56]
466.0	Acute bronchitis	Viral/Bacterial	1859 (20.14)	6556 (14.20)	<0.001	<0.001	1.52 [1.44 to 1.61]
460	Acute nasopharyngitis (common cold)	Viral	1737 (18.82)	7551 (16.36)	<0.001	<0.001	1.19 [1.12 to 1.26]
465.0	Acute laryngopharyngitis	Viral/Bacterial	243 (2.63)	976 (2.11)	0.002	0.018	1.25 [1.08 to 1.44]
466.03	Acute tracheobronchitis	Viral/Bacterial	345 (3.74)	990 (2.15)	<0.001	<0.001	1.77 [1.56 to 2.01]
466.1	Acute bronchiolitis	Viral	65 (0.70)	157 (0.34)	<0.001	<0.001	2.08 [1.53 to 2.79]
487	*Influenza*	Viral	515 (5.58)	1466 (3.18)	<0.001	<0.001	1.80 [1.62 to 2.00]
463	Acute tonsillitis	Bacterial	1918 (20.78)	7272 (15.75)	<0.001	<0.001	1.40 [1.33 to 1.48]
461.8	Sinusitis, other, acute	Bacterial	779 (8.44)	2056 (4.45)	<0.001	<0.001	1.98 [1.81 to 2.16]
483.8	Pneumonia due to other specified organism	Bacterial	234 (2.54)	739 (1.60)	<0.001	<0.001	1.60 [1.37 to 1.86]
Gastrointestinal System Infections							
558.94	Gastroenteritis	Viral/Bacterial	1825 (19.77)	6913 (14.98)	<0.001	<0.001	1.40 [1.32 to 1.48]
534.45	Gastritis due to *Helicobacter pylori*	Bacterial	627 (6.79)	1588 (3.44)	<0.001	<0.001	2.05 [1.86 to 2.25]
112.84	*Candidal* esophagitis	Fungal	33 (0.36)	21 (0.05)	<0.001	<0.001	7.88 [4.42 to 14.34]
007.1	*Giardiasis*	Parasitic	15 (0.16)	22 (0.05)	<0.001	0.004	3.41 [1.65 to 6.89]
070.7	Unspecified viral hepatitis C	Viral	25 (0.27)	82 (0.18)	0.069	0.283	1.53 [0.93 to 2.41]
V02.61	Carrier or suspected carrier of hepatitis B	Viral	24 (0.26)	72 (0.16)	0.038	0.175	1.67 [1.00 to 2.68]
127.4	*Enterobiasis*	Parasitic	105 (1.14)	436 (0.95)	0.092	0.348	1.21 [0.96 to 1.50]
Genitourinary Infections							
599.0	UTI—Urinary tract infection, site not specified	Bacterial	3681 (39.87)	12,478 (27.03)	<0.001	<0.001	1.79 [1.71 to 1.88]
614	PID—Pelvic inflammatory disease	Bacterial	276 (2.99)	445 (0.96)	<0.001	<0.001	3.17 [2.71 to 3.69]
Skin and Soft Tissue Infections							
682.90	Abscess NOS	Bacterial	375 (4.06)	1097 (2.38)	<0.001	<0.001	1.74 [1.54 to 1.96]
682.91	Cellulitis NOS	Bacterial	349 (3.78)	1079 (2.34)	<0.001	<0.001	1.64 [1.45 to 1.86]
686.9	Skin and subcutaneous tissue local infection, unspecified	Bacterial	113 (1.22)	314 (0.68)	<0.001	<0.001	1.81 [1.44 to 2.25]
684	Impetigo	Bacterial	87 (0.94)	284 (0.62)	0.001	0.007	1.54 [1.19 to 1.96]
675.11	Mastitis of breast	Bacterial	96 (1.04)	379 (0.82)	0.041	0.185	1.27 [1.00 to 1.59]
110.41	*Tinea pedis*	Fungal	1051 (11.38)	3357 (7.27)	<0.001	<0.001	1.64 [1.52 to 1.76]
110.32	*Tinea cruris*	Fungal	319 (3.46)	949 (2.06)	<0.001	<0.001	1.71 [1.49 to 1.94]
112	*Candidiasis*	Fungal	155 (1.68)	396 (0.86)	<0.001	<0.001	1.97 [1.63 to 2.39]
111.02	*Tinea versicolor*	Fungal	149 (1.61)	401 (0.87)	<0.001	<0.001	1.87 [1.54 to 2.27]
112.3	Candidiasis of skin and nails	Fungal	67 (0.73)	206 (0.45)	0.001	0.007	1.63 [1.22 to 2.16]
078.10	Viral warts, unspecified	Viral	259 (2.81)	937 (2.03)	<0.001	<0.001	1.39 [1.21 to 1.60]
Systemic Infections							
078.89	Viral diseases, other specified	Viral	1253 (13.57)	4587 (9.94)	<0.001	<0.001	1.42 [1.33 to 1.52]
079.841	Symptomatic novel COVID-19 infected patient	Viral	990 (10.72)	2956 (6.40)	<0.001	<0.001	1.76 [1.63 to 1.89]
078.5	*Cytomegaloviral disease [CMV]*	Viral	44 (0.48)	119 (0.26)	0.001	0.008	1.85 [1.28 to 2.64]
072.91	Parotitis infectious—Mumps—without mention of complications	Viral	13 (0.14)	20 (0.04)	0.002	0.013	3.25 [1.49 to 6.87]
041.84	Other specified bacterial infections	Bacterial	258 (2.80)	641 (1.39)	<0.001	<0.001	2.04 [1.76 to 2.37]
041.1	*Staphylococcus* infection	Bacterial	125 (1.35)	326 (0.71)	<0.001	<0.001	1.93 [1.56 to 2.38]
683	Acute lymphadenitis	Bacterial	64 (0.69)	129 (0.28)	<0.001	<0.001	2.49 [1.81 to 3.39]
075	Infectious mononucleosis—*Epstein–Barr infection* (viral)	Viral	22 (0.24)	58 (0.13)	0.015	0.088	1.90 [1.11 to 3.15]

ICD—International Classification of Diseases; FM—fibromyalgia; BH FDR—Benjamini–Hochberg False Discovery Rate; OR—Odds Ratio.

## Data Availability

Data are available upon reasonable request through the corresponding author.
